# New Tacrine Analogs as Acetylcholinesterase Inhibitors — Theoretical Study with Chemometric Analysis

**DOI:** 10.3390/molecules18032878

**Published:** 2013-03-04

**Authors:** Paweł Szymański, Robert Skibiński, Tadeusz Inglot, Marek Bajda, Jakub Jończyk, Barbara Malawska, Elżbieta Mikiciuk-Olasik

**Affiliations:** 1 Department of Pharmaceutical Chemistry and Drug Analyses, Medical University, Muszyńskiego 1, 90-151 Lodz, Poland; E-Mail: elzbieta.mikiciuk-olasik@umed.lodz.pl; 2 Department of Medicinal Chemistry, Pharmaceutical Faculty, Medical University of Lublin, Jaczewskiego 4, 20-090 Lublin, Poland; E-Mails: robert.skibinski@umlub.pl (R.S.); t.inglot@umlub.pl (T.I.); 3 Department of Physicochemical Drug Analysis, Chair of Pharmaceutical Chemistry, Faculty of Pharmacy, Jagiellonian University Medical College, 30-688 Krakow, Medyczna 9, Poland; E-Mails: marek.bajda@uj.edu.pl (M.B.); jakub.jonczyk@uj.edu.pl (J.J.); barbara.malawska@uj.edu.pl (B.M.)

**Keywords:** acetylcholinesterase inhibitors, chemometric analysis, docking, prediction of BBB penetration, Ames test simulation

## Abstract

Computer simulations constitute the basis of the design and discovery of new drugs. This approach is not only significant with regards to finding new structures, but also for selecting the molecules with the highest probability of being useful in the diagnostic process and treatment of numerous diseases. In our work, we used computational software to analyze 32 new acetylcholinesterase (AChE) inhibitors and formulate ADMET predictions. To understand the influence of the structure of our derivatives on binding mode, we docked all structures to the active site of AChE and assigned some pharmacophoric features. Finally, we undertook a chemometric analysis of all the compounds on the basis of FT-IR, which gave us the possibility of performing a fast categorization of the analyzed compounds and design compounds with similar structures.

## 1. Introduction

Alzheimer’s disease (AD) is a fatal neurodegenerative disease that affects elderly people. This disease is characterized by an irreversible degeneration of cholinergic neurons, which leads to an impairment of the cognitive functions. The result of this pathologic process is a decline in the cholinergic function of the central nervous system, which can only be visualized upon *post mortem* examination [1,2,3]. In the 1980s, it was confirmed that decreased level of choline acetyltransferase correlates with a decline in the mental status scores as described by Bartus [4]. This theory was the basis of one of the possible approaches to treating this disease. The treatment of AD is based on the improvement of cholinergic neurotransmission by inhibiting acetylcholinesterase (AChE) and butyrylcholinesterase (BuChE). AChE is an enzyme mainly found in the brain, muscles, erythrocytes and cholinergic neurons. BuChE occurs in lungs, heart, kidneys, liver and serum. In pathological states such as AD the level of BuChE builds up. Cholinesterase inhibitors reduce degradation of acetylcholine (ACh) in the synapse. Additionally, acetylcholinesterase inhibitors diminish the formation of amyloid fibrils and possess neuroprotective activity [5,6].

AD treatments have focused on reducing cognitive decline with cholinesterase inhibitors as the treatment of first choice, and currently five approved pharmaceuticals are available for this purpose: galantamine, rivastigmine, donepezil, tacrine, and memantine (an NMDA receptor antagonist).

In 1993, tacrine (9-amino-1,2,3,4-tetrahydroacridine, THA) was approved for clinical use by the FDA in the United States as an effective drug in the treatment of AD. Henceforth, the chemical structure of THA has been modified numerous times using various approaches. On occasion, the modifications pertain to the tetrahydro ring or placemnt of ligands in positions 6, 7 [7,8]. Several modifications concern position 9 in tacrine—these were performed via an introduction of a carbon chain with other ligands or a tacrine moiety (resulting in a bis-tacrine or tacrine hybrid) [9,10]. Typical analyses of new acetylcholinesterase inhibitors (AChEIs) are concerned with measuring their activity (Ellman’s method) as well as examining activity towards beta-amyloid and gamma-secretase. On the basis of these assays, the structure-activity relationship is calculated. Very often docking to the active site is performed, which explains the mechanism of binding with enzyme. Sometimes, other analytic tools are used as well.

**Figure 1:**
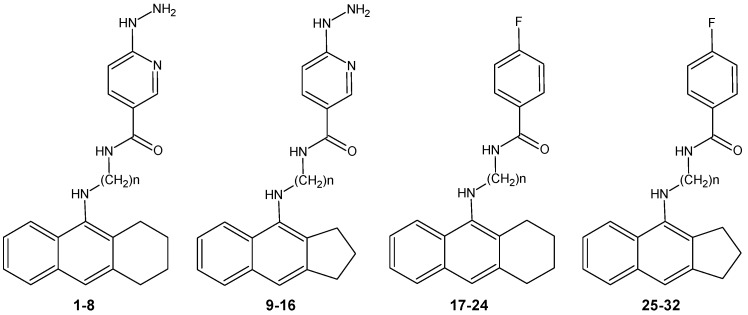
Analyzed compounds: tetrahydroacridine derivatives **1**–**8** and **17**–**24**; cyclopenta[b]quinoline derivatives **9**–**16** and **25**–**32** [11,12,13,14,15,16].

The aim of this study was a pharmacokinetic characterization of a library of cholinesterase inhibitors for selection of the most appropriate compounds for *in vivo* tests, as well as the creation of a pharmacophore model that could be useful for the design of novel potential inhibitors. In this paper, we present a theoretical study of 32 new acetylcholinesterase inhibitors (Figure 1) for which *in vitro* analyses towards AChE, BuChE and their selectivities were previously reported [11,12,13,14,15,16]. Our analysis concerns the computer simulation of the ADMET process, which is very important in selecting specific compounds for subsequent experiments on animals. Also, we recorded FT-IR spectra and on the basis of these results, prepared a chemometric analysis. Finally, we docked all compounds to acetylcholinesterase from the 2CKM crystal structure and found and assigned a number of pharmacophoric features.

## 2. Results and Discussion

### 2.1. Genotoxicity

**Figure 2:**
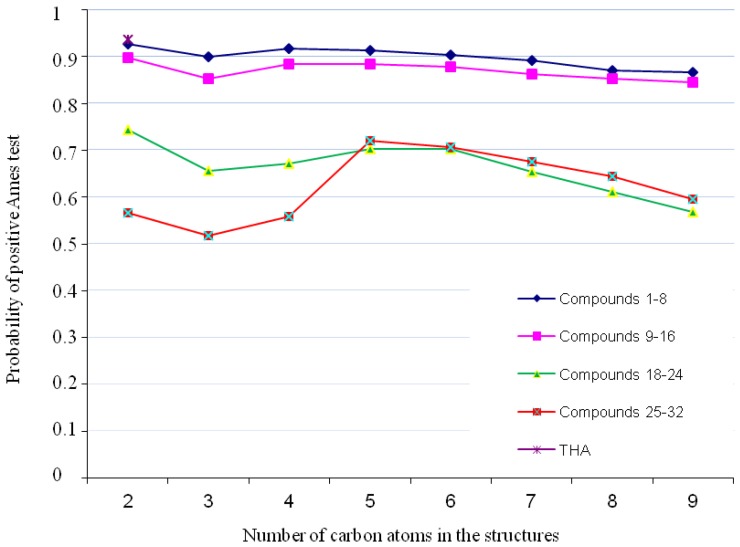
Genotoxicity profile for analyzed compounds.

For all compounds, we calculated the probability for a positive Ames test. Assessment of genotoxicity was performed using standardized data and a Percepta software model (which contains a database of 8,607 compounds). A model of the Ames test was used to compare our compounds with tacrine (THA)—a reference acetylcholinesterase inhibitor. Genotoxicity for all compounds was found to be lower or comparable to THA for which the value of Ames test is 0.94. The replacement of the 6-hydrazinenicotinic acid moiety with fluorobenzoic acid has a significant influence on decreasing the toxicity in this group of compounds as compounds with 6-hydrazinenicotinic acid moieties are more toxic. On the other hand, the change in the structure from 1,2,3,4-tetrahydroacridine to cyclopenta[b]quinoline, doesn’t appear to have any meaningful influence on toxicity (Figure 2).

**Figure 3:**
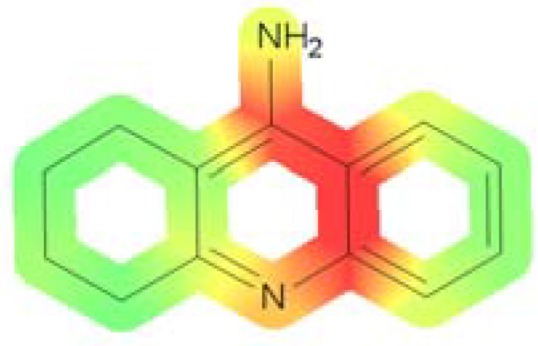
Visualization of the genotoxic properties for tacrine. Hazardous substructures (genotoxic fragments) are colored in red.

We identified the structural fragments potentially responsible for genotoxic effect [17]. Analysis of our compounds yielded a list of structural alerts. Tacrine was used as the baseline pattern (Figure 3). On this structure we observe color-coded atomic/fragment contributions which suggest potential genotoxicity—the green part is not involved in genotoxic activity and the red is responsible for genotoxic properties. Each of our compounds possessed a part of the tacrine structure with an aromatic amine. Aromatic amines exhibit genotoxic activity after being metabolized to hydroxylamines [18,19,20]. The case of all of our compounds was similar situation, in that the same part of the aromatic ring had a high probability to be toxic.

**Figure 4:**
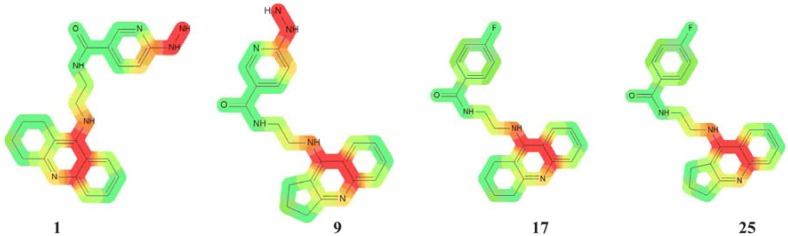
Different “hazardous” substructures were identified. Compounds with two carbon atoms in the chain from every group are presented.

Compounds **1**–**16** were found to be the most genotoxic. The structures of these compounds have the 6-hydrazinonicotinate moiety (Figure 4). It is known that unsubstituted, bonded heteroatoms are metabolized into various unstable radicals and other reactive substructures [21]. However, in the case of other compounds (compounds **17**–**32**) with 4-fluorobenzoic acid moieties, toxic activity was lower, suggesting that this moiety doesn’t have any influence on genotoxicity.

### 2.2. Cardiotoxicity

We next predicted for our compounds the probability of inhibiting the hERG potassium channel. We used a Global, Adjusted Locally According to Similarity (GALAS) modeling methodology (values of hERG inhibition are estimated automatically by the software module), which uses a comparison between cardiotoxicity and hERG channel at a clinically relevant concentration of K_i_ < 10 μM. Prediction of the reliability index (RI - assess the quality of the predictions) suggests that all compounds have a small affinity for the hERG channel. For all compounds, RI index was found to be at a threshold value of 0.3.

### 2.3. P-Glycoprotein Specificity

P-glycoprotein 1 (permeability glycoprotein) is also known as a multidrug resistance protein 1 (MDR1) or ATP-binding cassette sub-family B member 1 (ABCB1). P-gp is a well characterized transporter, which moves a variety of substrates across extra- and intracellular membranes. P-gp is extensively distributed, being expressed in the intestinal epithelium, hepatocytes, renal proximal tubular cells, adrenal glands, and capillary endothelial cells, which comprise the blood-brain and blood-testis barrier [22]. Therefore, predicting P-gp transport and inhibition is very important in many fields aiming to develop anti-cancer and CNS-active drugs [22].

P-gp inhibitor probability was found to be relatively low. In every group of compounds (Figure 1) we can observe that this value is increasing with the number of carbon atoms in the aliphatic chain. We notice for compound **24** the highest value of P-gp inhibitor probability and the lowest value is predicted for the compound **16**. Generally, compounds with a 4-fluorobenzoic acid moiety have higher values than compounds with 6-hydrazinonicotinate moieties. Next, we evaluated the probability of P-gp substrate to transport and eliminate compounds. In this case, molecules with long aliphatic chains in the structures have a higher probability of being a suitable P-gp substrate (similarly to P-gp inhibitor probability). Derivatives with 4 and 5 carbons in the aliphatic chain had the highest probability of being P-gp substrates. These calculations show that tetrahydroacridine derivatives with 6-hydrazinonicotinate moieties have higher P-gp substrate probability than cyclopenta[b]quinolines with florobenzoic acid moieties. Finally, compounds **3**, **4**, **11**, **12** should undergo a very rapid transport to the outside of the body (the highest value of P-gp substrate probability).

### 2.4. Blood Brain Barier (BBB) Penetration

We evaluated the blood-brain barrier (BBB) penetration potential of our compounds. Each compound was assessed whether it would permeate to the CNS [23]. Compounds were classified using a reliable theoretical model with a reasonable prediction of the rate and extent of BBB permeation. The values of the following three, automatically calculated quantitative parameters were used to classify the compounds as CNS permeable or non-permeable:

- rate of brain penetration (LogPS); this parameter depends on essential physicochemical properties such as lipophilicity, ionization constants, hydrogen bonding parameters, and molecular size;
- extent of brain penetration (LogBB); cumulative effect of drug binding to brain and plasma (LogBB is assumed to correspond to the passive transport across blood-brain barrier);
- brain/plasma equilibration rate (Log(PS*f_u,brain_)); f_u,brain_ – fraction unbound in plasma, calculated automatically [23].

**Figure 5:**
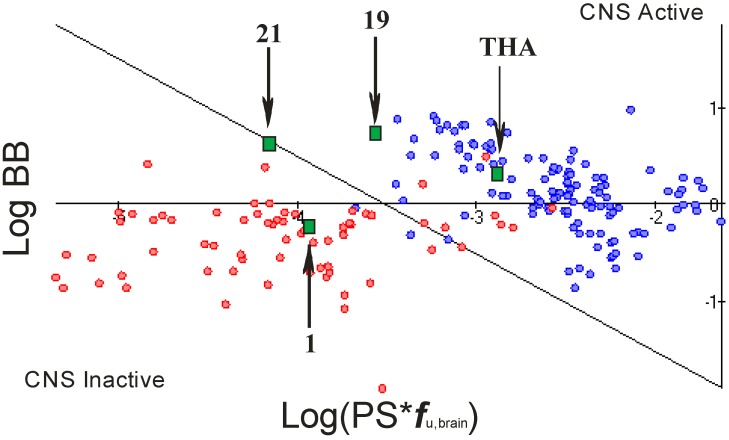
CNS activity plot showing known CNS-penetrating (blue points) and peripherally acting drugs (red points). The green quadrates denote some currently analyzed molecules correctly classified by our model as CNS active for compound **19** or CNS inactive for compound **1**.

Quantitative BBB transport parameters were automatically calculated for all 32 compounds and for THA as a reference. It can be observed that compounds **1**–**16** (compounds with 6-hydrazinenicotinate moieties) are CNS inactive. In the case of compounds **17**–**32** (compounds with fluorobenzoic acid moieties), only compounds **17**–**20** show a possibility of CNS activity. Compound **21** was found to possess a threshold value (Figure 5). Results of simulations for compounds **25**–**29** with cyclopenta[b]quinoline as the basic structure suggest the highest brain/plasma penetration.

We also calculated the topological polar surface area (TPSA). The major advantage of TPSA is that it can be used for prediction of drug transport properties such as BBB penetration. It is defined as the surface area (Å^2^) occupied by nitrogen and oxygen atoms and attached to them, polar hydrogens (strongly associated with hydrogen bonding capacity and polarity). 

Drugs that penetrate CNS should have lower polar surface areas than other kinds of molecules [24]. In the case of CNS, penetrating drugs are estimated at 60 Å^2^ to 90 Å^2^. The maximum PSA value for a molecule able to penetrate to the brain is around 90 Å^2^ [25,26]. In our study, compounds **1**–**16** have a very high level of TPSA, while the value for compounds **17**–**32** is 54 Å^2^. This is the group where we can look for compounds with brain affinity.

### 2.5. Distribution

In the analysis of new drugs, it is very important to know the basic pharmacokinetic parameters such as protein binding, volume of distribution, or affinity to serum albumin. We predicted some of these parameters to better characterize our compounds. For the estimation of binding of compounds to human plasma proteins, *e.g*., albumin, lipoproteins and alpha-1-acid glycoprotein we used the %PPB parameter as the cumulative percentage. In general, for all compounds **1**–**32** binding with proteins is very high in comparison with THA (78%). For compounds with short linkers (carbon chains) in the structures, it is around 91%–93% and for longer linkers with 8–9 carbon atoms in the chain, protein binding is nearly 99%.

To mark the human serum albumin affinity we used logK_a_^HSA^. We predicted this parameter for all our compounds. Compounds **1**–**16** (compounds with 6-hydrazinenicotinate moieties) have similar values of logK_a_^HSA^, around 3.5, which are slightly increased with carbon chain elongation. We obtained very interesting data for compounds **17**–**32** (compounds with fluorobenzoic acid moieties) as the value of logK_a_^HSA^ was bigger than that of HA. For tacrine, which was used as a reference compound, this value was 4.2, but for molecules **17**–**32** it was higher. For compound **17** it was 4.3 and the highest value was for compound **24** (5.06). Very often, the interactions between drugs and the human serum albumin can have a significant influence on the distribution volume and the elimination rate of drugs as a result of their binding to serum albumin [27].

The volume of distribution (Vd) is a theoretical volume that a drug would have to occupy, to provide the same concentration as in plasma. It is known that the human body is primarily (~70%) made up of water, therefore, this parameter depends on the physiologic properties of the body and the physiochemical properties of the drug. Our predicted value of Vd is expressed in liters per kg body weight and is similar for every group of our compounds. For our molecules, higher values of Vd correspond to compounds with a long carbon chain (8–9 carbon atoms) and these values are 10.6–18.8 L/kg. On the other hand, compounds with 2 or 3 carbon atoms in the linker were found to have 5.2–8.6 L/kg - similar to THA (5.4 L/kg). This result suggests that compounds are destined to be confined to the plasma, or liquid part of the blood. If the volume is higher, the compounds will be distributed throughout the blood (plasma and red blood cells).

### 2.6. EC_50_ and LC_50_

LD_50_ is an abbreviation for Lethal Dose 50%, which is the amount of the substance required to kill 50% of the test population per body weight (mg/kg). Currently, the U.S. Food and Drug Administration has begun to approve non-animal techniques for estimating LD_50_ in response to research animal cruelty concerns and the lack of validity/sensitivity of animal tests. In this analysis, we estimated LC_50_ for all compounds for fish in mg/L (ppm) at 96 h. Results suggest that the value of this parameter is smaller for compounds with long carbon chains. In other words, compounds with longer carbon chains are more toxic than compounds with 2 or 3 carbon atoms in the aliphatic linker. Compounds **1**–**8**, **9**–**16** have very high toxicity (low LD_50_ values) and this seems due to the presence of the 6-hydrazinenicotinate moiety which is toxic. We also estimated EC_50_ (half maximal effective concentration). Results and values are similar to LC_50_ and refer commonly to the measure of drug's potency, where 50% of its maximal effect is observed. For competition binding assays and functional antagonist assays, IC_50_ is the most common summary measure of the dose-response curve.

### 2.7. Docking Studies and Pharmacophore Search

**Figure 6:**
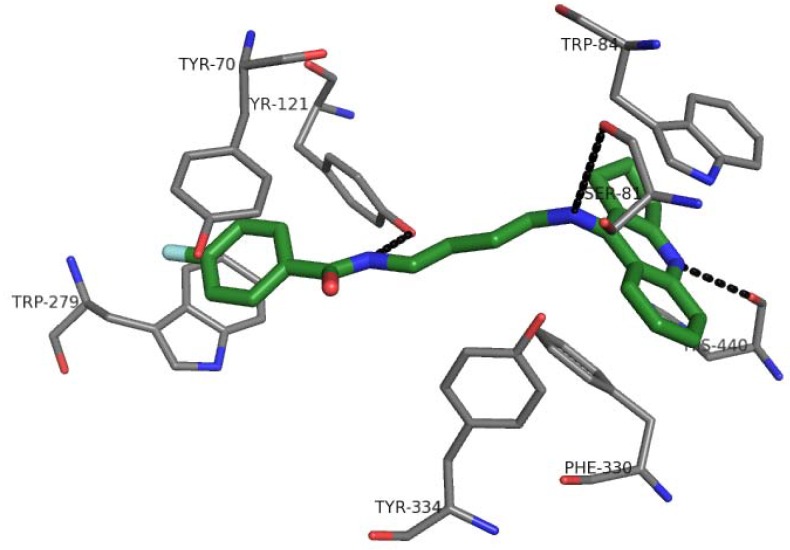
The binding mode of compound **27** into the active site of acetylcholinesterase.

The binding mode of novel compounds with acetylcholinesterase (AChE) was evaluated by a docking procedure. All ligands were divided into four groups, according to the differences in their structure (group **A**: compounds **1**–**8**, **B**: **9**–**16**, **C**: **17**–**24**, **D**: **25**–**32**). The analysis of binding mode was performed with reference to the inhibitors present inside each group and between groups. All 32 derivatives presented similar arrangement in the active gorge, interacting with catalytic and peripheral active site of acetylcholinesterase. The compounds belong to the dual-binding site inhibitors, which is very advantageous as they are able to influence numerous AChE functions. For example, these compounds are able to increase cholinergic transmission and inhibit the processing of Aβ through the PAS of the enzyme which interacted with oligomers and promoted their aggregation. All compounds presented the extended conformation. Tricyclic moieties, tetrahydroacridine or cyclopentaquinoline, were engaged in the formation of a characteristic sandwich due to π–π stacking with Trp84 and Phe330. The protonated form of tacrine or its analogue gave hydrogen bond with carbonyl group of His440. We did not find any significant differences between those moieties, with the exception that a cyclopentaquinoline created a smaller hydrophobic surface area. The linker was located in the middle of the gorge, where it created hydrophobic interactions, mainly with Tyr334. Its optimal length was 4–5 methylene groups (compounds **4**, **20**, **27** from series **A**, **C** and **D**, respectively) because this provided the best fit of the outermost aromatic fragments of the molecule to both active sites. The exception was series **B** and compound **16** with nine carbon atoms in the tether. The benzamide phenyl ring (compounds **17**–**32**) interacted with Trp279 and Tyr70, forming the other sandwich with the best fit for compounds **20** and **27**. It also created CH-π interactions with Tyr121. A similar situation was found in the case of series **A**. The pyridine ring also created a sandwich with Trp279 and Tyr70. The linker amine and amide groups created hydrogen bonds with carbonyl group of Ser81 and hydroxyl substituent in Tyr121, respectively. The strongest H-bonds between amide group and OH Tyr121 were formed for compounds with 4–5 carbon linkers, especially for derivative **27**. Other inhibitors were not able to provide such a beneficial arrangement and the hydrogen bond was weaker or was not created. Hydrazine in compound **16** (series **B**) interacted by H-bond with carbonyl group of Asp276 backbone; consequently, pyridine ring was shifted and it wasn’t able to create classical π–π stacking. It was engaged in some hydrophobic interactions. The fluorine substituent (series **C** and **D**) created hydrophobic interactions mainly with side chain of Ile175 and also with Trp279 and Tyr70. The binding mode of the most active compound **27** is presented in Figure 6.

**Figure 7:**
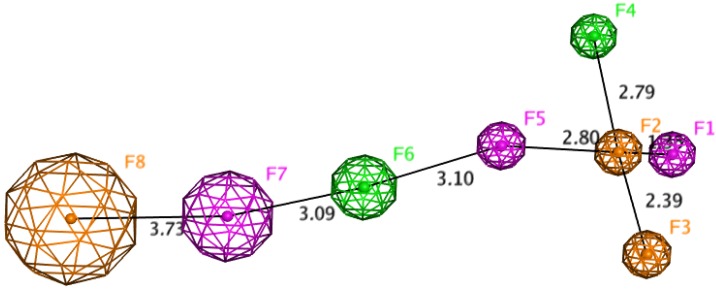
Pharmacophore model for the novel class of acetylcholinesterase inhibitors (meaning of colors: orange – aromatic feature, green – hydrophobic, pink – H-bond donor; radius of feature represents precision of assignment).

The top-ranked poses for each ligand were the starting point for creating the pharmacophore model. It was assumed that docking solutions represented bioactive conformations. These conformations were compared to one another to find and assign a number of pharmacophoric features, enabling generation of eight-point pharmacophore. This model was composed of three aromatic, two hydrophobic, and three H-bond donor features. The first five elements (F1, F5 – donors, F2, F3 – aromatic, F4 – hydrophobic) were precisely assigned and corresponded to the tricyclic moiety (two aromatic and one cycloalkyl rings) with two hydrogen bond donors. They were responsible for good affinity to catalytic active site of acetylcholinesterase. The F6 hydrophobic feature conditioned interactions in the middle of the active gorge. The last two elements, F7 (donor) and F8 (aromatic), provided binding with the peripheral anionic site. Their assignment was found to be of limited accuracy; therefore the radius of the tolerance sphere of these features was larger. The distances between all features are presented in Figure 7. The knowledge of a pharmacophore model might be useful in further studies on acetylcholinesterase inhibitors. It can be used in the design of novel active compounds or in virtual screening. Currently, several pharmacophore models for cholinesterase inhibitors were described, and among them there is a model for a series of tacrine derivatives [28]. This model consists of six features: two hydrogen-bond donors (HBDs), one hydrogen-bond acceptor (HBA), two aromatic rings (RA) and one hydrophobic feature (H). Comparing presented above and our model, we can state that both contain six common elements, however our model possesses two additional features and all distances between important elements were measured and presented. This comparison indicates that different methodologies led to valuable results for further design of potential inhibitors. Our pharmacophore enables to optimize novel molecules as well as to perform screening of huge databases in relatively short period of time.

### 2.8. Chemometric Analysis

Two multivariate chemometrics methods, principal component analysis (PCA) and hierarchical cluster analysis (HCA), were used for chemical characterization of the analyzed compounds based on their ATR-FT-IR spectra. PCA identifies the natural clusters in the data set by the decorrelation of the variables, and converts them into the linear combinations called principal components. 

**Figure 8:**
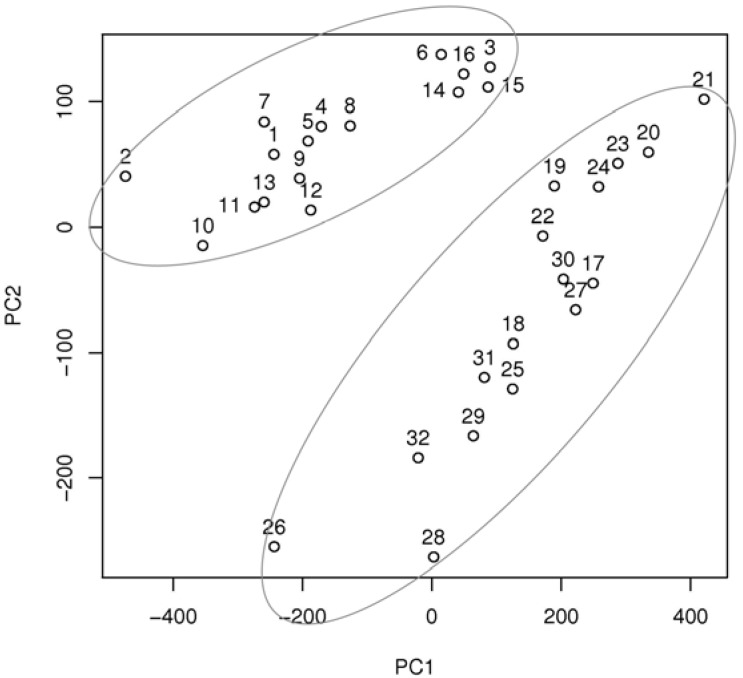
Principal component analysis (PCA) of ATR-FT-IR spectra of 32 analyzed AChE inhibitors.

The first two principal components (PC1 and PC2) explain the maximum available overall variances and can be used to make a two-dimensional map. This map enables the analysis of similarities between the spectra. As shown in Figure 8, the analyzed compounds were categorized into two significant groups. The first group (compounds **1**–**16**), represents compounds with hydrazinyl substituents, while the other group represents fluoro derivatives of the analyzed substances (compounds **17**–**32**). In this analysis, PC1 explained 60.9% of the total variance, whereas the first two principal components explained 75.3% of the variance. Principal component analysis can be used for the fast characterization of the analyzed AChE inhibitors into two main groups, in this case: hydrazine and fluoro derivatives.In order to perform a more specific categorization of the analyzed compounds, hierarchical cluster analysis was also used. HCA helps to identify the similarities between the spectra using the distance between spectra and aggregation algorithms. A typical presentation of the hierarchical approach is a tree (dendrogram) with individual elements at one end, and a single cluster containing every element at the other end. In this study, hierarchical cluster analysis was performed with the use of Minkowski distance with p = 10 (the power of the Minkowski distance). 

**Figure 9:**
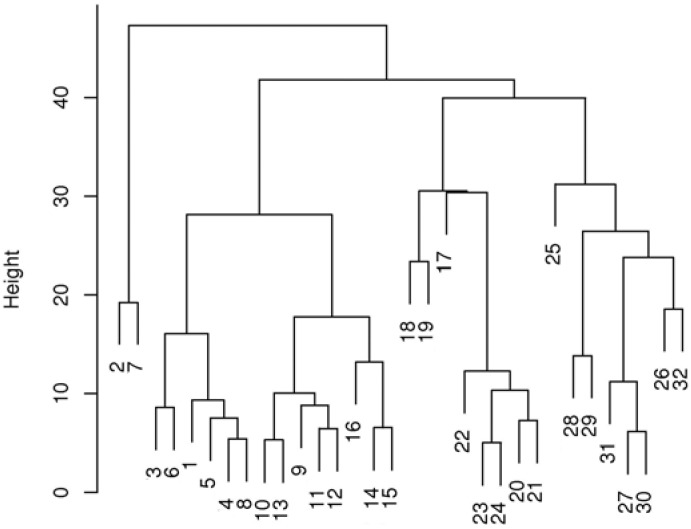
The dendrogram obtained using Minkowski distance for ATR-FT-IR spectra of 32 analyzed AChE inhibitors.

As shown in Figure 9, the HCA method divided the substances into five main groups, with a significant chemical rule being observed in this case.

The first (compounds **2** and **7**) and the second (compounds **1**, **3**–**6** and **8**) groups represent hydrazinyl-substituted tetrahydroacridine derivatives. The third group (compounds **9**–**16**) is a cluster of hydrazinyl-substituted dihydrocyclopentaquinoline derivatives. The fourth (compounds **17**–**24**) and the fifth groups (compounds **25**–**32**) represent fluorotetrahydroacridine and fluorodihydrocyclo-pentaquinoline derivatives, respectively. Although compounds **2** and **7** are clustered separately from compounds **1**–**8**, HCA is an appropriate method for investigating the similarity between IR spectra of analyzed AChE inhibitors. The similarity between the spectra is very often correlated with a similar chemical structure; fast categorization of the analyzed compounds into four main structural groups was possible in this case.

## 3. Experimental

Computer predictions of absorption, distribution, metabolism, excretion and toxicity (ADMET) properties were prepared with ACD/Percepta 14.0.0. The 2D structures and SMILES codes were drawn in ACD/Labs.

### 3.1. Genotoxicity

The genotoxicity module allows for better determination of the mutagenic potential of compounds. We used the following separate tools to estimate the potential hazard and the risk of genotoxicity:

- the Ames test to provide the mutagenic potential of new compounds
- a knowledge-based system in identifying and visualizing hazardous fragments of structures.

These two tools enable the identification of potentially hazardous compounds and assessment of genotoxicity by researchers. For predicting standardized Ames genotoxicity, we used well-known public databases, containing more than 8,500 compounds, namely, Chemical Carcinogenesis Research Information (CCRIS) and Genetic Toxicology Data Bank (GENE-TOX). On the basis of this data, we calculated the probability of a positive Ames test of our compounds. In the next step, we used the genotoxicity hazards module to identify and visualize structural fragments potentially involved in genotoxic activity [17,29].

### 3.2. Cardiotoxicity

The hERG potassium ion channel inhibition and analysis of this process is the basis in pharmacological safety research of new drugs beceause inhibition of this channel may lead for example to arrhythmia, which may occasionally have fatal results due to subsequent ventricular fibrillation [30]. Therefore, early identification of cardiotoxicity prevents costly failures and potential safety issues in drugs design. In ACD/Percepta we used the hERG Inhibition module to estimate the potential of hERG-related cardiotoxicity of potential drugs. The built model consisted of experimental hERG inhibition data for 663 compounds collected from two types of assays. Electrophysiological patch-clamp assay (512 compounds) constituted the first assay. Inhibitors were defined as compounds with hERG current inhibition IC_50_ < 10 μM; and non-inhibitors: IC_50_ ≥ 10 μM. The second assay consisted of radio ligand displacement assay (161 compounds). Inhibitors: *Ki* < 0.5 μM; and non-inhibitors: *Ki* > 100 μM. Generally, the used modules calculated the probability of the compound being a hERG inhibitor, where IC_50_ was lower than the cut-off value of 10 μM. The data were verified and converted to binary representation to obtain the following values: 1 – inhibitors, 0 – non-inhibitors [30].

### 3.3. P-Glycoprotein Specificity

P-glycoprotein (P-gp) is an energy-dependent efflux transporter that extrudes compounds from a large variety of cells. P-gp is found in the epithelial cells of enterocytes along the luminal side of the cell. In the case of orally ingested drugs, molecules have to pass through the enterocyte to enter the blood. P-gp can transfer the molecules and carry them back to the luminal side of the cell; thus, preventing drug molecules from joining the systemic circulation, and effectively limiting bioavailability. In most cases, potential drugs that are substrates or inhibitors of P-gp are undesirable and must be, consequently, excluded from the next step of drug design. Thanks to P-gp specificity modules we can establish whether a compound is a P-gp substrate or inhibitor. Predicting the probability of a substance being a P-gp substrate or inhibitor is calculated by using a fully searchable database of 2,290 structures. This data contains a collection of P-gp substrates (>1,000 compounds) and inhibitors (>1,500 compounds). The predictive model calculates the probability for the analyzed compounds and provides information on:·P-gp substrate, High affinity P-gp substrate, P-gp inhibitor or Potent P-gp inhibitor with *Ki* < 1 μM [31].

### 3.4. Blood Brain Barier (BBB) Penetration

Transport across the blood-brain barrier (BBB) is the most important feature of new drugs intended to have activity in the central nervous system (CNS). To estimate if the given compound will penetrate the brain to a sufficient level for activity in the CNS, kinetic and thermodynamic characteristics of drug transport across the BBB should be estimated [32].

BBB transport parameters were automatically calculated according to the presence or absence of CNS activity. It was expressed by the rate (log*PS*) and extent (log*BB*) of brain penetration. These parameters were automatically calculated by physicochemical descriptors: for calculating log*PS* (Permeability-Surface), software used the most important descriptors as a lipophilicity, ionization parameters (acid and base pKa), as well as hydrogen bonding potential and molecular size. On the other hand, for calculating log*BB* (the steady-state distribution ratio between brain tissue and plasma) was used unbound fraction in plasma. All descriptors were calculated automatically. In this way, we obtained predictions whether a given compound is permeable enough to exhibit activity in the CNS. BBB provides reliable and easily interpretable predictions of both rate and extent of BBB permeation by passive diffusion (expressed as Log*PS* and Log*BB* constants respectively) [33].

To prepare the plot of CNS activity, we used the parameter PS as a -*F* * (1 - e*-Kin/F*); it is defined from the kinetic equation of capillary transport and is equal to the influx rate constant *Kin* corrected for blood flow rate in cerebral micro capillaries denoted as *F*. However, the extent of brain penetration (log*BB*) is determined by the ratio of total drug concentrations in tissue and plasma at steady-state conditions (log*BB* = log(*cbrainSS/cbloodSS)* in further simulations we predicted the unbound fraction in the brain tissue (*fu, brain*) and brain/plasma equilibration rate log (*PS* * *fu, brain) -* combination of permeation rate and fraction unbound in brain [34].

### 3.5. Distribution

We performed the pharmacokinetic analysis of our compounds using the distribution module. Firstly, we predicted the extent of plasma protein binding, and then calculated the percentage of freely circulating compound, which was pharmacologically active. We calculated the volume of distribution for a quick estimation of the distribution of compounds between plasma and body tissue. In this module we calculate other parameters as a %PPB (the cumulative percentage of a compound bound to human plasma proteins, such as albumin) and Log K_a_^HSA^. %PPB values represent the overall fraction of the bound drug in human plasma; this parameter was established on the basis of almost 1s500 compounds [35,36]. Log K_a_^HSA^ (the human serum albumin affinity constant), where log K_a_^HSA^ represents the compounds’ affinity constant to human serum albumin, was used to calculate %PPB. Experimental data, used in model originated from direct chromatographic determination of binding strength to that particular protein (human serum albumin): log K_a_^HSA^ = log([LA]/[L][A]); where, [LA] – the concentration of ligand bound to albumin, [L] – free ligand, [A] – concentration of free albumin. Values of the volume of distribution (Vd) constituted the last parameter of the distribution, showing the effect of physicochemical properties (LogP and ionization) in the body. Supplementary distribution/Vd module calculates the apparent volume of distribution of drugs in the human body expressed in liters per kg body weight (L/kg). When this parameter is high then the drug is distributed in tissue (*i.e.*, not in plasma).

### 3.6. EC_50_ and LC_50_

EC_50_ and LC_50_ were calculated using ECOSAR Version 1.10 software (US Environmental Protection Agency). This software is based on more than 150 SAR (structure-activity relationship) models and has been developed for more than 50 chemical classes based on measured test data. This data have been submitted by industry. It estimates the aquatic toxicity of chemicals possessing a structure similar to that of the chemicals for which aquatic toxicity has previously been measured *in vitro*. The analyzed compounds have been developed based on ECOSARs models datasets [37].

### 3.7. Molecular Modeling

Corina on-line (Molecular Networks) was used for creating three-dimensional structure of the ligands. Checking the atom types, adding hydrogen atoms, and assignment of Gasteiger-Marsili were performed with Sybyl 8.0 (Tripos). Acetylcholinesterase from the 2CKM crystal structure was prepared for docking in the following way: all histidine residues were protonated at Nε, the hydrogen atoms were added, and ligand and water molecules were removed. The binding site was defined as all amino acid residues within 10 Å from the reference ligand – bis-(7)-tacrine. Docking was performed with GoldSuite 4.1 (CCDC). A standard set of genetic algorithms with population size 100, number of operations 100,000, and clustering with a tolerance of 1Å were applied. As a result, 10 ligand poses, sorted by GoldScore function value, were obtained. The results were visualized by PyMOL 0.99rc6 (DeLano Scientific LLC). The docking poses with the highest score for each ligand were used for the pharmacophore generation. They were superimposed and then some features were assigned with MOE2009.10 (CCG).

### 3.8. ATR-FT-IR and Multivariate Chemometric Analysis

The IR spectra of the analyzed compounds were recorded in attenuated total reflectance (ATR) mode using a Thermo Scientific Nicolet 6700 Fourier transform infrared spectrometer equipped with a smart ITR diamond adapter (Madison, Wisconsin USA). The assay was performed in the range 650–4,000 cm^−1^ at spectral resolution of 2 cm^−1^ and the number of the sample scans was set at 32, meaning that each spectrum represented 32 co-added scans of the analyzed sample. The background (air) spectrum was also recorded in the same conditions. Spectral data was acquired with Omnic software and all computations were performed in the GNU R environment in version 2.14.2.

## 4. Conclusions

Several different computational approaches have been used for design of new cholinesterases inhibitors [38]. Usually computational strategies for acetylcholinesterase inhibitors are related to structure - activity relationship (SAR) and quantitative structure - activity relationship (QSAR) analyses or include virtual screening method [39]. Very often we can observe analysis of the selected bioactive conformations from docking procedure of new compounds which are used to establish a three dimensional model (sometimes expressed by Comparative Molecular Field Analysis - CoMFA) [40]. In this paper, we have presented a new kind of theoretical study of the AChEI. We decided on the prediction of basic ADMET parameters for the structures obtained by modification of a well-known AChE inhibitor – tacrine. In this study, we presented theoretical analyses of 32 previously obtained derivatives of tetrahydroacridine and cyclopenta[b]quinoline as inhibitors of acetylcholinesterase. We defined ADMET processes for our compounds on the basis of computer simulations. We calculated some parameters such as genotoxicity and cardiotoxicity. Those parameters suggest that derivatives of cyclopenta[b]quinoline have better values that warrant further analysis. The other parameters, specifically P-glycoprotein and BBB, show that compounds with cyclopenta[b]quinoline and fluorobenzoic acid substructures have the greatest probability of being active in the CNS. These compounds are most likely to remain in the organism for long enough time to reach therapeutical targets. We showed that derivatives of cyclopenta[b]quinoline were characterized by smaller genotoxicity in comparison with classic tetrahydroacridine derivatives. However, AChEI which have smaller values of EC_50_, LC_50_ are more toxic. This is observed for compounds with long carbon chains in conjunction with 6-hydrazinenicotinate moieties. When we compare prediction results of ADME parameters with AChE activity we can suggest that the best compound for *in vivo* testing is compound **27** (*N*-[4-(2,3-dihydro-1*H*-cyclopenta[b]quinolin-9-ylamino)butyl]-4-fluorobenzamide), which shows the highest activity against AChE and it is a derivative of cyclopenta[b]quinoline with a fluorobenzoic acid moiety. The choice of compound **27** remains in accordance with our predictions of ADME parameters.

Finally we performed chemometric analysis and prepared a pharmacophore model, which might be useful in further studies on acetylcholinesterase inhibitors. Results of chemometric analysis can be routinely applied to future data in order to predict the same quality parameters for similar groups of new compounds before making UV analysis. On the other hand, the pharmacophore model may be used in the design of novel active compounds or in the virtual screening, giving the possibility of a fast categorization of the analyzed compounds. It can be use to check a lot of virtual libraries with structures of new compounds *in silico* and to find compounds with high probability to be acetylcholinesterase inhibitors.

## Supplementary Materials

Supplementary materials can be accessed at: http://www.mdpi.com/1420-3049/18/3/2878/s1.
