# Supplementary material for: New Tacrine Analogs as Acetylcholinesterase Inhibitors — Theoretical Study with Chemometric Analysis
Source: Molecules. 2013 Mar 4;18(3):2878–94. doi: 10.3390/molecules18032878 (PMC6270554; doi:10.3390/molecules18032878)
Supplement: Supplementary file 1 [file molecules-18-02878-s001.pdf]

Supplementary Materials

Figure S1. Structure of the analysed compounds.

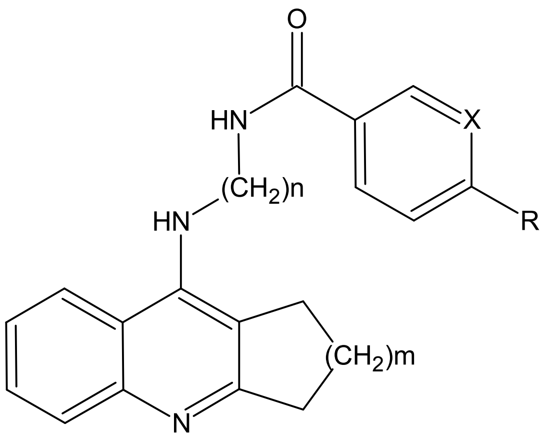

Table 1. Chemical names of analysed compounds with acetylcholinesterase activity.

No	Compound	X	R	n	m	Activity (AChE inhibition IC_{50}, nM)
1	6-Hydrazino-*N*-[2-(1,2,3,4-tetrahydroacridin-9-ylamino)ethyl]nicotinamide	N	$NHNH_2$	2	2	5.6 ± 0.4
2	6-Hydrazino-*N*-[3-(1,2,3,4-tetrahydroacridin-9-ylamino)propyl]-nicotinamide	N	$NHNH_2$	3	2	3.4 ± 0.2
3	6-Hydrazino-*N*-[4-(1,2,3,4-tetrahydroacridin-9-ylamino)butyl]nicotinamide	N	$NHNH_2$	4	2	3.4 ± 0.3
4	6-Hydrazino-*N*-[5-(1,2,3,4-tetrahydroacridin-9-ylamino)pentyl]nicotinamide	N	$NHNH_2$	5	2	2.7 ± 0.2
5	6-Hydrazino-*N*-[6-(1,2,3,4-tetrahydroacridin-9-ylamino)hexyl]nicotinamide	N	$NHNH_2$	6	2	4.4 ± 0.4
6	6-Hydrazino-*N*-[7-(1,2,3,4-tetrahydroacridin-9-ylamino)heptyl]nicotinamide	N	$NHNH_2$	7	2	3.1 ± 1.9
7	6-Hydrazino-*N*-[8-(1,2,3,4-tetrahydroacridin-9-ylamino)octyl]nicotinamide	N	$NHNH_2$	8	2	7.2 ± 1.9
8	6-Hydrazino-*N*-[9-(1,2,3,4-tetrahydroacridin-9-ylamino)nonyl]nicotinamide	N	$NHNH_2$	9	2	3.7 ± 1.9
9	6-Hydrazino-*N*-[2-(2,3-dihydro-1*H*-cyclopenta[b]quinolin-9-ylamino)ethyl]nicotinamide	N	$NHNH_2$	2	1	31.5 ± 1.9
10	6-Hydrazino-N-[3-(2,3-dihydro-1H-cyclopenta[b]quinolin-9-ylamino)propyl]nicotinamide	N	$NHNH_2$	3	1	19.3 ± 1.1
11	6-Hydrazino-*N*-[4-(2,3-dihydro-1*H*-cyclopenta[b]quinolin-9-ylamino)butyl]nicotinamide	N	$NHNH_2$	4	1	22.4 ± 1.7
12	6-Hydrazino-*N*-[5-(2,3-dihydro-1*H*-cyclopenta[b]quinolin-9-ylamino)pentyl]nicotinamide	N	$NHNH_2$	5	1	7.8 ± 2.2
13	6-Hydrazino-*N*-[6-(2,3-dihydro-1*H*-cyclopenta[b]quinolin-9-ylamino)hexyl]nicotinamide	N	$NHNH_2$	6	1	41.6 ± 1.0
14	6-Hydrazino-*N*-[7-(2,3-dihydro-1*H*-cyclopenta[b]quinolin-9-ylamino)heptyl]nicotinamide	N	$NHNH_2$	7	1	17.6 ± 0.8

Table 1. *Cont.*

No	Compound	X	R	n	m	Activity (AChE inhibition IC50, nM)
15	6-Hydrazino-*N*-[8-(2,3-dihydro-1*H*-cyclopenta[b]quinolin-9-ylamino)octyl]nicotinamide	N	$NHNH_2$	8	1	5.2 ± 1.4
16	6-Hydrazino-*N*-[9-(2,3-dihydro-1*H*-cyclopenta[b]quinolin-9-ylamino)nonyl]nicotinamide	N	$NHNH_2$	9	1	3.7 ± 0.5
17	4-fluoro-*N*-[2-(1,2,3,4-tetrahydroacridin-9-ylamino)ethyl]-benzamide	C	F	2	2	8.2 ± 0.2
18	4-fluoro-*N*-[3-(1,2,3,4-tetrahydroacridin-9-ylamino)propyl]-benzamide	C	F	3	2	8.1 ± 0.3
19	4-fluoro-*N*-[4-(1,2,3,4-tetrahydroacridin-9-ylamino)butyl]-benzamide	C	F	4	2	7.8 ± 0.2
20	4-fluoro-*N*-[5-(1,2,3,4-tetrahydroacridin-9-ylamino)pentyl]-benzamide	C	F	5	2	5.3 ± 0.2
21	4-fluoro-*N*-[6-(1,2,3,4-tetrahydroacridin-9-ylamino)hexyl]-benzamide	C	F	6	2	24.3 ± 0.8
22	4-fluoro-*N*-[7-(1,2,3,4-tetrahydroacridin-9-ylamino)heptyl]-benzamide	C	F	7	2	22.8 ± 1.4
23	4-fluoro-*N*-[8-(1,2,3,4-tetrahydroacridin-9-ylamino)octyl]-benzamide	C	F	8	2	5.6 ± 0.4
24	4-fluoro-*N*-[9-(1,2,3,4-tetrahydroacridin-9-ylamino)nonyl]-benzamide	C	F	9	2	13.4 ± 4.0
25	*N*-[2-(2,3-dihydro-1*H*-cyklopenta[b]quinolin-9-ylamino)ethyl]-4-fluorobenzamide	C	F	2	1	1.1 ± 0.1
26	*N*-[3-(2,3-dihydro-1*H*-cyklopenta[b]quinolin-9-ylamino)propyl]-4-fluorobenzamide	C	F	3	1	10.8 ± 0.5
27	*N*-[4-(2,3-dihydro-1*H*-cyklopenta[b]quinolin-9-ylamino)butyl]-4-fluorobenzamide	C	F	4	1	0.5 ± 0.1
28	*N*-[5-(2,3-dihydro-1*H*-cyklopenta[b]quinolin-9-ylamino)penthyl]-4-fluorobenzamide	C	F	5	1	5.8 ± 0.3
29	*N*-[6-(2,3-dihydro-1*H*-cyklopenta[b]quinolin-9-ylamino)hexyl]-4-fluorobenzamide	C	F	6	1	153.0 ± 5.2
30	*N*-[7-(2,3-dihydro-1*H*-cyklopenta[b]quinolin-9-ylamino)heptyl]-4-fluorobenzamide	C	F	7	1	2.8 ± 0.2
31	*N*-[8-(2,3-dihydro-1*H*-cyklopenta[b]quinolin-9-ylamino)octyl]-4-fluorobenzamide	C	F	8	1	5.2 ± 0.9
32	*N*-[9-(2,3-dihydro-1*H*-cyklopenta[b]quinolin-9-ylamino)nonyl]-4-fluorobenzamide	C	F	9	1	5.5 ± 0.2
33	9-amino-1,2,3,4-tetrahydroacridine (THA)	-	-	-	2	5.5 ±1.0

Table 2. Values of probability of Ames test, P-gp inhibitor, P-gp substrate and values of quantitative parameters for BBB predictions.

n	No	Probability of positive Ames test	P-gp inhibitor probability	P-gp substrate probability	LogPS	LogBB	Fraction unbound in brain ($f_{u,brain}$)	Log(PS*$f_{u,brain}$)
2	1	0.93	0.32	0.63	−3.02	−0.24	0.12	−3.94
3	2	0.90	0.46	0.71	−2.95	−0.11	0.09	−3.99
4	3	0.92	0.42	0.82	−2.81	0.10	0.06	−4.03
5	4	0.91	0.42	0.82	−2.66	0.19	0.04	−4.07
6	5	0.90	0.52	0.75	−2.54	0.29	0.03	−4.12
7	6	0.89	0.58	0.73	−2.36	0.53	0.01	−4.34
8	7	0.87	0.64	0.75	−2.43	0.58	0.01	−4.52
9	8	0.87	0.61	0.76	−2.53	0.48	0.01	−4.66
2	9	0.90	0.17	0.55	−3.32	−0.48	0.25	−3.92
3	10	0.85	0.34	0.55	−3.22	−0.36	0.19	−3.95
4	11	0.88	0.27	0.74	−3.00	−0.08	0.10	−3.98
5	12	0.88	0.28	0.70	−2.84	−0.05	0.07	−4.01
6	13	0.88	0.35	0.61	−2.65	0.12	0.04	−4.06
7	14	0.86	0.43	0.53	−2.41	0.35	0.02	−4.18
8	15	0.85	0.44	0.62	−2.36	0.39	0.01	−4.26
9	16	0.85	0.46	0.64	−2.41	0.56	0.01	−4.50
2	17	0.74	0.69	0.65	−1.57	0.51	0.02	−3.34
3	18	0.66	0.66	0.67	−1.55	0.69	0.01	−3.44
4	19	0.67	0.70	0.78	−1.56	0.73	0.01	−3.56
5	20	0.70	0.70	0.81	−1.72	0.65	0.01	−3.86
6	21	0.70	0.74	0.74	−1.95	0.64	0.01	−4.13
7	22	0.65	0.75	0.75	−2.37	0.46	0.01	−4.57
8	23	0.61	0.78	0.77	−2.80	0.41	0.01	−5.00
9	24	0.57	0.79	0.76	−2.90	0.24	0.01	−5.10
2	25	0.57	0.28	0.49	−1.55	0.28	0.02	−3.33
3	26	0.52	0.42	0.44	−1.55	0.32	0.01	−3.40
4	27	0.56	0.50	0.72	−1.56	0.49	0.01	−3.60
5	28	0.72	0.57	0.67	−1.56	0.44	0.01	−3.58
6	29	0.71	0.58	0.47	−1.67	0.51	0.01	−3.80
7	30	0.67	0.63	0.49	−2.02	0.50	0.01	−4.21
8	31	0.64	0.67	0.55	−2.32	0.46	0.01	−4.52
9	32	0.60	0.70	0.59	−2.68	0.37	0.01	−4.88
−	33	0.94	0.08	0.21	−1.93	0.30	0.11	−2.89

P-gp - P-glycoprotein 1 (permeability glycoprotein); **LogPS** - rate of brain penetration; **LogBB** - extent of brain penetration; $\mathbf{f_{u.brain}}$ - fraction unbound in brain.

Table 3. Values of parameters: the cumulative percentage of compound bound to human plasma proteins, the human serum albumin affinity, three basis parameters of chemical structure,volume of distribution, lethal dose and half maximal effective concentration.

n	No	%PPB	LogKa(HSA)	Vd (L/kg)	TPSA	No. of Hydrogen Bond Donors	No. of Hydrogen Bond Acceptors	LC50 96 h fish mg/L(ppm)	EC50 96 h green alge mg/L(ppm)
2	1	93.14	3.64	6.06	104.96	5	7	52.81	30.63
3	2	92.98	3.66	6.05	104.96	5	7	19.84	12.67
4	3	92.33	3.49	6.90	104.96	5	7	7.44	5.23
5	4	94.01	3.55	8.18	104.96	5	7	2.79	2.16
6	5	94.85	3.66	9.38	104.96	5	7	1.04	0.89
7	6	96.39	3.77	10.75	104.96	5	7	0.39	0.37
8	7	96.92	3.83	11.75	104.96	5	7	0.15	0.15
9	8	97.80	3.87	13.48	104.96	5	7	0.05	0.06
2	9	91.62	3.39	5.19	104.96	5	7	140.37	73.95
3	10	91.82	3.18	5.18	104.96	5	7	52.81	30.63
4	11	91.38	3.11	5.91	104.96	5	7	19.84	12.67
5	12	94.00	3.30	7.00	104.96	5	7	7.44	5.23
6	13	94.93	3.38	8.02	104.96	5	7	2.79	2.16
7	14	96.18	3.50	9.20	104.96	5	7	1.04	0.89
8	15	96.89	3.56	10.55	104.96	5	7	0.39	0.37
9	16	97.03	3.61	11.53	104.96	5	7	0.15	0.15
2	17	94.48	4.85	8.56	54.02	2	4	0.15	0.15
3	18	93.68	4.78	8.63	54.02	2	4	0.34	0.32
4	19	94.72	4.69	9.77	54.02	2	4	0.13	0.13
5	20	96.76	4.76	11.46	54.02	2	4	0.05	0.06
6	21	97.13	4.94	13.05	54.02	2	4	0.02	0.02
7	22	98.16	4.99	14.85	54.02	2	4	0.01	0.01
8	23	98.39	5.02	16.91	54.02	2	4	0.00	0.00
9	24	98.91	5.06	18.79	54.02	2	4	0.00	0.00
2	25	96.82	4.56	7.31	54.02	2	4	2.42	1.87
3	26	97.07	4.61	7.28	54.02	2	4	0.91	0.77
4	27	97.21	4.27	8.24	54.02	2	4	0.34	0.32
5	28	97.40	4.51	9.66	54.02	2	4	0.13	0.13
6	29	97.63	4.63	11.00	54.02	2	4	0.05	0.06
7	30	97.93	4.73	12.52	54.02	2	4	0.02	0.02
8	31	98.18	4.76	14.25	54.02	2	4	0.01	0.01
9	32	98.54	4.82	16.22	54.02	2	4	0.00	0.00
-	33	78.48	4.22	5.42	38.91	2	2	13.90	8.61

%PPB - the cumulative percentage of compound bound to human plasma proteins (such as albumin, alpha1-acid glycoprotein and others); **Ka(HSA)** - the human serum albumin affinity constant; **Vd** - the volume of distribution; **TPSA** - the topological polar surface area; **LC50** - lethal dose 50%. which is the amount of the substance required to kill 50% of the test population per body weight; **EC50** - the term half maximal effective concentration refers to the concentration of a drug, where 50% of its maximal effect is observed.
